# Combination L-Glutamine with Gemcitabine and Nab-Paclitaxel in Treatment-Naïve Advanced Pancreatic Cancer: The Phase I GlutaPanc Study Protocol

**DOI:** 10.3390/biomedicines11051392

**Published:** 2023-05-08

**Authors:** Jun Gong, Arsen Osipov, Jeremy Lorber, Mourad Tighiouart, Albert K. Kwan, Hayato Muranaka, Rasaq Akinsola, Sandrine Billet, Abrahm Levi, Anser Abbas, John Davelaar, Neil Bhowmick, Andrew E. Hendifar

**Affiliations:** 1 Department of Medicine, Samuel Oschin Comprehensive Cancer Institute, Cedars-Sinai Medical Center, Los Angeles, CA 90048, USA; 2 Biostatistics and Bioinformatics Research Center, Samuel Oschin Comprehensive Cancer Institute, Cedars-Sinai Medical Center, Los Angeles, CA 90048, USA

**Keywords:** pancreatic cancer, metastatic, L-glutamine, gemcitabine, nab-paclitaxel, chemotherapy, clinical trial

## Abstract

Advanced pancreatic cancer is underscored by progressive therapeutic resistance and a dismal 5-year survival rate of 3%. Preclinical data demonstrated glutamine supplementation, not deprivation, elicited antitumor effects against pancreatic ductal adenocarcinoma (PDAC) alone and in combination with gemcitabine in a dose-dependent manner. The GlutaPanc phase I trial is a single-arm, open-label clinical trial investigating the safety of combination L-glutamine, gemcitabine, and nab-paclitaxel in subjects (*n* = 16) with untreated, locally advanced unresectable or metastatic pancreatic cancer. Following a 7-day lead-in phase with L-glutamine, the dose-finding phase via Bayesian design begins with treatment cycles lasting 28 days until disease progression, intolerance, or withdrawal. The primary objective is to establish the recommended phase II dose (RP2D) of combination L-glutamine, gemcitabine, and nab-paclitaxel. Secondary objectives include safety of the combination across all dose levels and preliminary evidence of antitumor activity. Exploratory objectives include evaluating changes in plasma metabolites across multiple time points and changes in the stool microbiome pre and post L-glutamine supplementation. If this phase I clinical trial demonstrates the feasibility of L-glutamine in combination with nab-paclitaxel and gemcitabine, we would advance the development of this combination as a first-line systemic option in subjects with metastatic pancreatic cancer, a high-risk subgroup desperately in need of additional therapies.

## 1. Introduction

Systemic chemotherapy remains the preferred treatment for pancreatic cancer that is advanced or unresectable. Combination regimens, including 5-fluorouracil (5-FU)/leucovorin, irinotecan, and oxaliplatin (FOLFIRINOX), or gemcitabine and nab-paclitaxel, have been recently established as first-line standards in patients with preserved performance status (PS) [1]. For patients with poor PS, gemcitabine alone has remained a standard cytotoxic agent for treating metastatic pancreatic cancer [1]. Following the failure of gemcitabine, the administration rates of second-line and third-line chemotherapy in metastatic pancreatic cancer have approximated 45% and 21%, respectively, of 117 patients with metastatic pancreatic cancer [2]. These values reinforce the importance of optimizing first-line chemotherapy, given the aggressive nature of advanced pancreatic cancer that is underscored by progressive therapeutic resistance and a dismal 5-year survival rate of 3% [3]. Thus, we investigate the safety of combining L-glutamine with gemcitabine and nab-paclitaxel as a novel therapeutic approach for treatment-naïve patients with unresectable or metastatic pancreatic ductal adenocarcinoma (PDAC.) In the GlutaPanc trial, which is an open-label, single-arm, single-institution phase I clinical trial, we initially seek to establish the phase II recommended dose (RP2D) for the combination of L-glutamine, gemcitabine, and nab-paclitaxel in these patients.

Preclinical investigations with human pancreatic ductal adenocarcinoma (PDAC) cells have shown that oncogenic *KRAS* mutations reprogram glutamine metabolism through an alternative pathway that fuels the tricarboxylic acid (TCA) cycle and promotes tumor growth [4,5]. Specifically, mutant *KRAS* upregulates several enzymes, resulting in the transport of glutamine-derived aspartate into the cytoplasm that maintains cellular redox for adenosine triphosphate (ATP) production through increasing the ratio of reduced nicotinamide adenine dinucleotide phosphate (NADPH) to nicotinamide adenine dinucleotide phosphate (NADP^+^) and the generation of pyruvate. Moreover, preclinical studies have demonstrated that oncogenic *Kras* alters glucose metabolism in PDAC, resulting in enhanced glycolysis that drives glycolytic intermediates into anabolic pathways important for proliferation [6]. Specifically, mutant *Kras* regulates key enzymes involved in glucose metabolism, which increases glycolytic flux that drives glucose intermediates into (1) the hexosamine biosynthesis pathway, important for post-translational protein modification and glycolipid and proteoglycan synthesis, and (2) the nonoxidative pentose phosphate pathway, important for nucleic acid synthesis, without affecting the trafficking of glycolytic metabolites into the TCA cycle.

Unfortunately, early endeavors to antagonize glutamine metabolism in PDAC preclinical models as a therapeutic strategy have largely failed [7]. Instead, a growing body of evidence suggests that glutamine deprivation activates IκB kinase β (IKKβ) and p53, which promotes cancer cell adaptation to glutamine depletion [8,9]. Furthermore, in PDAC in vitro and in vivo models, glutamine deprivation results in the accumulation of microRNA-135 in a p53- and reactive oxygen species-dependent manner, which also contributes to cancer cell adaptation and survival [10]. Interestingly, one preclinical study demonstrated decreased cancer cell viability when cells were cultured in higher concentrations of glutamine and the systemic agent PLX4032 (i.e., vemurafenib, a mutant BRAF^V600E^ kinase inhibitor) [11]. Recently, several groups have shown glutamine supplementation to elicit antitumor effects in vivo while achieving elevated glutamine concentrations in the blood and intratumorally to generate tumor response, compared to controls. For example, melanoma-bearing mouse models treated with oral glutamine exhibited significant reductions in tumor volume with corresponding increases in serum glutamine concentrations, compared to mice fed with control diets [12]. In vivo PDAC mouse models have corroborated the antitumor effects of supplementing oral glutamine at 5 mg/kg per day doses of 100% glutamine, compared to controls, where mice treated with glutamine supplementation demonstrated significantly prolonged survival compared to the control [13].

The mechanisms for these paradoxical findings that glutamine supplementation, rather than glutamine deprivation, can elicit antitumor effects, remain unknown. We are in a unique position to investigate the potential metabolic changes elicited by oral L-glutamine with or without chemotherapy for the first time in human subjects with metastatic PDAC. *Kras*-driven metabolic reprogramming in PDAC has been shown to affect a multitude of metabolic pathways, as described earlier. Based on preclinical evidence of metabolic pathways altered by oncogenic *Kras* in PDAC, we hypothesize that L-glutamine supplementation may further alter the metabolic pathways initially reprogrammed by *KRAS* to sustain tumor proliferation.

Therefore, we will perform serial plasma metabolomics in human PDAC subjects with metastatic disease treated with L-glutamine with or without chemotherapy. We hope to attain mechanistic insights into the possible anticancer properties of L-glutamine that may rely on metabolic pathways altered by mutant *KRAS* and to uncover potential associations between metabolic profiles associated with therapeutic response (or lack thereof). Additionally, L-glutamine has been implicated in promoting gut health [14]. Oral glutamine has long been used, with evidence that it can mitigate chemotherapy- and radiation therapy-induced gastrointestinal toxicities as well [15]. We are therefore proposing to also evaluate changes in the stool microbiome before and after glutamine supplementation, utilizing the 1-week L-glutamine lead-in phase of this study. Here, we will investigate the impact of L-glutamine on the gut microbiome composition of our subjects with advanced PDAC prior to the introduction of standard chemotherapy. Rates of gastrointestinal side effects to chemotherapy will be associated with changes in the stool microbiota.

The GlutaPanc phase I trial will investigate the recommended phase II dose of combining gemcitabine and nab-paclitaxel with L-glutamine as a first-line treatment in therapy-naïve patients with unresectable or metastatic pancreatic cancer. We hypothesize that the addition of L-glutamine sensitizes patients with metastatic PDAC to gemcitabine-based chemotherapy. The novelty of this study is that we will be the first to conduct a dose-finding study of the only U.S. Food and Drug Administration (FDA)-approved oral L-glutamine therapy in combination with standard chemotherapy for the first-line treatment of metastatic pancreatic cancer. Additionally, we will be among the first to evaluate metabolomics and the gut microbiome before and after a lead-in phase of L-glutamine alone, to gain mechanistic insight into the impact of glutamine supplementation on metabolic and stool microbiome profiles in subjects with pancreatic cancer.

The purpose of the study is to examine the safety of combination therapy of gemcitabine and nab-paclitaxel with L-glutamine as a first-line systemic therapy for treatment-naïve subjects with unresectable or metastatic pancreatic cancer. The primary objective is to establish the recommended phase II dose (RP2D) for the combination of gemcitabine, nab-paclitaxel, and L-glutamine in these patients. Secondary objectives include characterizing the tolerability and preliminary efficacy of the regimen in this cohort. Lastly, exploratory objectives include (1) performing plasma metabolomics in longitudinal samples throughout the course of the study to measure changes in multiple metabolic pathways that may be altered by L-glutamine in order to identify potential mechanistic insights and associations with study treatment, and (2) evaluating changes in the stool microbiome from available stool samples before and after the L-glutamine lead-in, to analyze its impact on promoting gut health as an additional potential mechanism of benefit.

**Figure 1:**
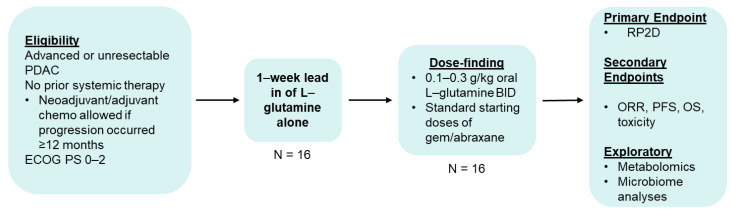
Study Schema of the GlutaPanc trial. A single-institution, open-label, single-arm, non-randomized, phase I clinical trial investigating the feasibility of combination L-glutamine, gemcitabine, and nab-paclitaxel in treatment-naïve, unresectable or metastatic pancreatic cancer. The primary objective is to determine the recommended phase II dose (RP2D) of combination L-glutamine, gemcitabine, and nab-paclitaxel in this patient population. PDAC, pancreatic ductal adenocarcinoma; ECOG PS, Eastern Cooperative Oncology Group (ECOG) performance status; BID, twice daily; ORR, overall response rate; PFS, progression-free survival; OS, overall survival.

The GlutaPanc trial is an unblinded, single-arm, therapeutic interventional trial. An overall perspective of the study schema and timeline of events is illustrated by Figure 1 and Table 1, respectively.

## 2. Materials and Methods

### 2.1. Eligibility Criteria and Screening

During the recruitment phase, patients eligible for the trial must initially fulfill all inclusion and exclusion criteria, which are summarized in Table 2 and Table 3, respectively.

Informed consent from subjects must be obtained prior to assessments for determining study eligibility. Assessments obtained as per standard of care may be used to determine study eligibility even if carried out before the informed consent process. Screening procedures must be performed within 4 weeks of the 7-day L-glutamine lead-in or within 5 weeks of Day 1, unless otherwise stated. The screening procedures are summarized in Table 1 and Table 4.

### 2.2. Study Design

#### 2.2.1. Lead-In Phase

The dose-finding portion of this study for the combination of gemcitabine, nab-paclitaxel, and L-glutamine will be preceded by an intended 7-day administration of L-glutamine alone (0.1, 0.2, or 0.3 g/kg oral twice daily), which is consistent with recent phase III data supporting this approach [16]. This 7-day lead-in phase of L-glutamine administration will allow plasma metabolites to be measured at baseline and following glutamine monotherapy timepoints and stool microbiome analyses, prior to the addition of chemotherapy. The exact length of the lead-in phase is at the treating investigator’s discretion, with a 3–7-day window permitted for the lead-in phase. Patients who cannot complete a minimum of 3 days of L-glutamine alone during the lead-in phase may be replaced, in line with the investigator’s judgment. After the lead-in period, oral compliance for L-glutamine is considered to be 85%. Less than 85% compliance is considered a deviation, reportable to the Institutional Review Board (IRB). No pre-medication is needed with L-glutamine administration. Supportive care medication such as anti-emetics may be given, based on physician judgment. A dosing diary and instructions will be provided for each enrolled subject, and review by study staff is required at each clinical visit. L-glutamine is supplied and will be delivered by Emmaus Medical, Inc. (Torrance, CA, USA).

#### 2.2.2. Dose-Finding Phase

This phase I study is designed to determine the maximum tolerated dose (MTD) using 5 dose levels, as depicted in Table 5. The MTD is defined as the dose level wherein the probability of dose-limiting toxicities (DLTs) at the MTD is *θ* = 0.33. We will use an extension of the escalation with the overdose control (EWOC) algorithm to design the trial [17]. The first patient will receive study drug at dose level 0, and the next patient’s dose will be decided according to the EWOC algorithm. At any time during the trial, a patient may be enrolled into the trial if there are no more than 2 patients with unresolved DLT status in the trial. For example, if 3 patients under treatment did not experience DLT but have not finished their cycle 1 therapy, then another patient cannot be enrolled into the trial.

One treatment cycle of the combination of gemcitabine, nab-paclitaxel, L-glutamine will consist of L-glutamine continuous oral daily dosing along with gemcitabine and nab-paclitaxel intravenous (IV) on days 1, 8, and 15 (every 28-day cycle), each given according to the dosing scheme (Table 5). A cycle will be defined around the initiation of gemcitabine and nab-paclitaxel (i.e., Day 1 of every 28-day cycle of gemcitabine and nab-paclitaxel) given in the outpatient setting. Evaluation of the RP2D and DLTs will be based on the first 28 days or 4 weeks (i.e., Cycle 1) of study therapy. The dose level closest to the median of the posterior distribution of the MTD will establish the RP2D. DLTs are defined as grade ≥3 adverse events (AEs) that are drug-related and occur during the first 4 weeks (i.e., Cycle 1) of treatment. Such DLTs include the following: grade 4 thrombocytopenia of any duration or grade 3 thrombocytopenia associated with a grade 3 hemorrhage, grade 4 (life-threatening) anemia, grade ≥3 febrile neutropenia, absolute neutrophil count (ANC) < 100 mcL for ≥3 days, ANC < 500 mcL for ≥7 days, any grade 3–4 toxicity that is non-hematologic in origin considered clinically significant by the PI or treating investigator, and any grade 3 toxicity that is non-hematologic in origin lasting for more than 72 h (with the exception of fatigue and alopecia and grade 3 diarrhea, nausea, and vomiting that resolves within 5 days).

Post-treatment imaging and follow-up will be performed 8 weeks after initiating therapy on cycle 1, day 1 (C1D1). Discontinuation from the study for AEs deemed unacceptable will require subject follow-up until AE stabilization or until the AE is resolved. The beginning of a subsequent cycle can be delayed, depending on toxicities of the prior cycle.

The administration of other cancer therapies, either investigational or commercial, unless specified below, are not allowed.

### 2.3. Study Agents

#### 2.3.1. L-Glutamine

L-glutamine is currently the only FDA-approved clinical-grade glutamine as an oral powder administered twice daily based on body weight to lessen acute sickle cell disease complications, based on a randomized phase III trial enrolling 230 subjects with sickle cell anemia or sickle β^0^-thalassemia [18]. Proposed doses of oral L-glutamine in this study are based on this only FDA-labeled indication for L-glutamine powder (Endari^®^) to date, as per phase III trial data and the preceding phase II study [18,19,20]. The glutamine dose levels will be twice-daily oral doses of 0.1, 0.2, and 0.3 g/kg (rounded to the nearest 5 g, with a max limit of 15 g twice daily (30 g/day) per any given dose level), consumed each day at the same time until confirmed progressive disease (PD), intolerance, or any discontinuation criteria are met.

The dose-limiting feature of oral glutamine is its administration; the solubility of glutamine is only 3.6% at 23 °C [21]. As a result, doses of >15 g have historically required over 400 mL of fluid and constituted a significant burden to cancer patients, who often have anorexia and would experience greater discomfort (nausea) with such continuous daily intake while on chemotherapy [21,22,23]. For this reason, the maximum daily dose of L-glutamine will be capped at the FDA-recommended upper limit of 15 g twice daily (30 g per day).

The FDA-approved doses of L-glutamine now permit administration of the powder in 240 mL of fluid per dose, which translates to about 500 mL of fluid intake daily [19]. As per FDA recommendations, each glutamine dose should be immediately mixed with 8 oz. (240 mL) of a beverage at cold or room temperature. Glutamine can also be consumed with 4 oz. (120 mL) to 6 oz. (177 mL) of food such as applesauce or yogurt, before ingestion [19]. L-glutamine is packaged in 5 g packets as a crystalline white powder in paper-foil-plastic laminate. Complete dissolution of the powder is not required for administration. If a dose of L-glutamine is accidentally skipped, the dose will be taken at the next scheduled dose at the regular time. A skipped dose will not be repeated at a later time. If vomiting occurs following a dose of L-glutamine, additional or doubling subsequent doses will not be allowed. Instead, L-glutamine will be resumed at the next scheduled dose.

The most common adverse reactions (incidence > 10%) from L-glutamine treatment in the earlier-mentioned phase III trial involving subjects with sickle cell anemia or sickle β^0^-thalassemia have included nausea, constipation, abdominal pain, headache, cough, extremity pain, back pain, and chest pain, although these were all AEs reflective of a study population with sickle β^0^-thalassemia or sickle cell anemia [18,19]. Moreover, across 47 prospective trials investigating enteral glutamine in >1600 adult patients (including those treated with radiotherapy and chemotherapy), glutamine was well-tolerated, without any definitive evidence of drug interactions or compromise in antitumor efficacy [16,18,20,21,22,23,24,25,26,27,28,29,30,31,32,33,34,35,36,37,38,39,40,41,42,43,44,45,46,47,48,49,50,51,52,53,54,55,56,57,58,59,60,61,62,63,64]. We therefore anticipate that L-glutamine will be safe at full FDA-labeled doses when combined with standard doses of gemcitabine/nab-paclitaxel, as proposed in Table 5. No dosing modifications and treatment delays have been recommended in the FDA package insert.

#### 2.3.2. Gemcitabine

Dosing levels for gemcitabine and nab-paclitaxel are based on standard dose levels as recommended by the FDA-labeled indication for this combination in metastatic PDAC [65]. Three doses for gemcitabine at 600, 800, and 1000 mg/m^2^ 30-min intravenous (IV) infusion on days 1, 8, and 15, and three doses for nab-paclitaxel at 75, 100, and 125 mg/m^2^ 30 min IV infusion on days 1, 8, and 15 (every 28-day cycle) will be explored in this study. Dose modifications and treatment delay guidelines for gemcitabine and nab-paclitaxel are described in Table 6, Table 7 and Table 8.

Under the FDA-labeled indication in untreated metastatic pancreatic cancer, gemcitabine can be given as a 30 min IV infusion at 1000 mg/m^2^ on days 1, 8, and 15 following nab-paclitaxel IV 30 min infusion at 125 mg/m^2^ on days 1, 8, and 15, every 28-day cycle [65]. The most commonly observed (incidence ≥ 20%) treatment-related AEs from gemcitabine and nab-paclitaxel in treated subjects with pancreatic cancer have included fatigue, neutropenia, alopecia, peripheral neuropathy, peripheral edema, pyrexia, diarrhea, nausea, vomiting, rash, decreased appetite, and dehydration [65].

#### 2.3.3. Nab-Paclitaxel

Nab-paclitaxel is given (Table 5) at 75, 100, or 125 mg/m^2^ 30 min IV infusion on days 1, 8, and 15 (every 28-day cycle) until confirmed PD, intolerance, or any discontinuation criteria are met. Nab-paclitaxel is to be given first, before gemcitabine. Pre-medication for nab-paclitaxel is permitted, as per institutional standard practice. Grade 1–2 hypersensitivity reactions have occurred on days of nab-paclitaxel administration, consisting of shortness of breath (1%) and hypotension, flushing, chest pain, and arrhythmia (all <1%) [65]. In patients who have had prior hypersensitivity reactions to nab-paclitaxel, premedication may be needed. Those with prior experience of a severe hypersensitivity reaction to nab-paclitaxel should not be rechallenged with nab-paclitaxel, and will undergo study removal.

#### 2.3.4. Prohibited Medications

The following concomitant medications are prohibited: any investigational agent; systemic corticosteroid therapy exceeding prednisone or the equivalent of 10 mg/day (except for pre-medication, treatment of study drug-related AEs, and as an anti-emetic agent prior to chemotherapy; topical, inhaled, and intranasal corticosteroids are allowed); other immunosuppressive or immunomodulating agents (e.g., cyclophosphamide, methotrexate, azathioprine, and tumor necrosis factor-*α* blockers); other chemotherapy or anticancer treatment not stated in the protocol; and any enteral (tube) feeding, parenteral nutrition, or other nutritional supplementation.

To date, L-glutamine has not been shown to interact with any other concomitant medications. Gemcitabine has shown little or no impact on the pharmacokinetics (clearance and half-life) of nab-paclitaxel, and nab-paclitaxel has shown little or no effect on gemcitabine pharmacokinetics. Nab-paclitaxel is catalyzed by CYP2C8 and CYP3A4, and treating investigators should be cautioned when giving nab-paclitaxel concomitantly with medicines known to induce or inhibit either CYP2C8 or CYP3A4. Subjects on strong CYP2C8 or CYP3A4 inhibitors or inducers should discontinue at least one week prior to starting nab-paclitaxel.

## 3. Study Protocol

### 3.1. Dose Modification and Treatment Delay Guidelines

Toxicity will be evaluated using version 5.0 of the National Cancer Institute (NCI) Common Terminology Criteria for Adverse Events (CTCAE). The frequency of toxicities per organ system will be tabulated, using descriptive statistics. Any patient who receives any amount of the study drugs will be deemed evaluable for toxicity.

Doses can be delayed or modified in the event of an AE or at the physician’s discretion. Adverse events will be collected and reviewed at every patient visit. During the DLT assessment period, patients will be evaluated for DLTs within 1 week (±7 days) after the last dose of gemcitabine/nab-paclitaxel, if doses are delayed. If there is more than a 2-week delay between doses (all doses must be received within 4 weeks), the patient is not evaluable for DLTs (but they will continue to follow the protocol-directed procedures).

To appropriately assess toxicity and possible DLTs during treatment, patients will be seen in clinic by a practitioner every week, or per the investigator’s discretion, with blood work drawn as per the study calendar. Therapy is to be given as long as the subject has no evidence of disease progression and meets the criteria for treatment as defined below.

After the DLT is complete, the current cycle may be repeated when the patient meets the following criteria: platelet count ≥100,000 cells/mm^3^, absolute neutrophil count (ANC) ≥1500 cells/mm^3^, and a non-hematologic toxicity must have resolved to ≤Grade 1. In the event that L-glutamine is held, due to toxicity related to L-glutamine, gemcitabine and nab-paclitaxel may continue as long as the patient meets the criteria for retreatment. At the discretion of the investigator, L-glutamine may be continued in the event that gemcitabine and/or nab-paclitaxel is held, due to toxicity. If L-glutamine is permanently discontinued, the patient will be taken off the study and may continue standard chemotherapy at the discretion of the investigator. If gemcitabine and nab-paclitaxel is permanently discontinued, the patient will be taken off the study.

Supportive medications or treatments (e.g., acetaminophen or diphenhydramine) may be administered as deemed necessary by the treating investigator, to provide adequate prophylactic or supportive care. Best supportive care (including antibiotics, growth factor support, optimal symptom control, correction of metabolic disorders, and pain management (including palliative radiotherapy, etc.)) should be used when necessary for all patients, so long as all eligibility criteria are still fulfilled.

### 3.2. Safety Assessments

Adverse events will be assessed through the following stepwise approach: (1) identify the AE type using the NCI CTCAE, version 5.0; (2) grade the AE using the NCI CTCAE, version 5.0; (3) determine the AE relatedness to the protocol therapy, using the attribution categories of definite (clearly related to study treatment), probable (likely related), possible (may be related), unlikely (doubtfully related), and unrelated (clearly NOT related to study treatment); and (4) Determine the prior experience of the AE.

Step 3 includes all AEs that occur during the last 30 days of the study treatment dose. Any event occurring more than 30 days after the last study treatment dose and which is attributable (possibly, probably, or definitely) to the study drug(s) must also be accordingly reported.

Unanticipated problems are defined by all of the following: (1) unexpected (in terms of severity, nature, frequency) based on (a) the study procedures described in the protocol, and (b) the subject population characteristics studied; (2) related, or possibly related, to research participation (“possibly related” refers to the fact that the event may have been caused by the research procedures, with a reasonable possibility); and (3) the suggestion that a greater risk of harm (including physical, economic, psychological, or social harm) than was previously known to a subject or group of subjects (including study subjects, research personnel, or those not directly involved in the current study) is presented by this research.

All subjects experiencing an AE, irrespective of its relationship to the study treatment, will be followed until the AE resolves or the signs or symptoms that comprise the AE return to baseline, any lab abnormalities have returned to baseline, there is a satisfactory explanation other than the study treatment for the changes observed, or death.

The PI must be notified by study staff or co-investigators ≤ 24 h after learning of any serious adverse events (SAEs), irrespective of attribution, occurring during study treatment or ≤30 days of the last study drug administration. SAEs deemed related to the protocol and on-study deaths will be reported to the Samuel Oschin Comprehensive Cancer Institute Data and Safety Monitoring Committee (SOCCI DSMC) within 24 h of awareness.

The Cedars-Sinai Medical Center (CSMC) IRB must be notified within 10 business days of “any unanticipated problems involving risk to subjects or others”, including any confidentiality breach that may involve subject risk or risk to others and any subject complaint that cannot be resolved by the PI or that indicates an unanticipated risk.

All SAEs will be forwarded to the sponsor in the same time frame as FDA regulatory reporting: ≤7 calendar days of knowledge of the event for fatal or life-threatening unexpected, suspected serious adverse events; and no longer than 15 calendar days for non-life threatening, non-fatal unexpected, suspected AEs.

### 3.3. Duration of Study Participation

All screening procedures are required within 4 weeks of the 3–7-day L-glutamine lead-in phase, or within 5 weeks of Cycle 1 Day 1. The DLT assessment of this study will be the first cycle, over 28 days. The patients will then continue on treatment until PD or until any of the following occur: unacceptable AEs; inter-current illness that prevents further study treatment; experiencing DLT(s), which precludes resuming treatment with dose reduction, due to unfavorable risk–benefit ratio (for the first 28-day cycle) in the opinion of the principal investigator; subject wishes to withdraw from the study; or changes in the condition of the subject that render the subject unacceptable for further study treatment, as per the investigator’s judgment. In the absence of the aforementioned criteria, subjects may continue with study therapy at the investigator’s discretion.

### 3.4. Duration of Follow-Up

Patients will undergo an end-of-treatment (EOT) assessment within 30 days after removal from treatment. Subjects removed from treatment for unacceptable AEs will be monitored until stabilization or resolution of the AE, and will then continue to be monitored for the duration of the study, as per protocol. After the EOT assessment, follow-up will involve an every-3-month phone call or medical chart review to collect survival data for 24 months.

### 3.5. Removal of Subjects from Study

Subjects can be removed from study treatment and/or the trial at any time as per their own request, or they may be removed at the investigator’s discretion for behavioral, administrative, or safety reasons. Subjects who withdraw from the study treatment prior to starting study intervention (Cycle 1 Day 1) will be replaced. Reasons for discontinuation will be documented, and are summarized in Table 9.

### 3.6. Dose-Finding Phase

This dose-finding study explores three different dosages for gemcitabine (600, 800, and 1000 mg/m^2^ IV 30 min infusion on days 1, 8, and 15, respectively), nab-paclitaxel (75, 100, and 125 mg/m^2^ IV over 30 min, on days 1, 8, and 15, respectively), and L-glutamine oral powder (0.1, 0.2, and 0.3 g/kg oral twice daily, rounded to nearest 5 g, with an upper limit of 15 g, twice daily (30 g/day) for any dose level). L-glutamine will be given 1 week (±1 day) prior to initiation of gemcitabine and nab-paclitaxel. Our sample size will not be based on the power of one study. Rather, upon enrollment of a total of 16 patients into the DLT assessment cohort, RP2D will be assessed by EWOC, an adaptive Bayesian design, using the dosing schema proposed in Table 5.

For the purpose of deriving the operating characteristics of the study, we will assume that successive cohorts of one patient will be treated, and the dose level for the first patient will be dose level 0 (Table 5). Based on all available data, the dose for each subsequent subject will be determined, such that the probability that the dose exceeds the MTD is equal to a pre-specified value *α*. In this trial, we will start at *α* = 0.4 and increase *α* in increments of 0.05 until *α* = 0.5; the value represents a compromise between the toxic side effects and therapeutic aspect of the agent. No dose skipping will be permitted. The trial will be terminated if the posterior probability is that the probability that DLT at dose level −2 exceeds *θ* = 0.8 or more.

Upon study completion, the RP2D will be estimated as the median of the marginal posterior distribution of the MTD, rounded to the nearest discrete dose level. The computation of the dose to be administered to each patient and the estimate of the MTD will be carried out with statistical analysis programs R and JAGS.

We evaluated the design operating characteristics of this algorithm for the true value of the MTD under three scenarios. Table 10 illustrates various summary statistics, including the percent of MTD recommendation using 1000 trial replicates, average percent of DLTs across all trials, percent of trials with an excessive DLT rate, and percent of patients allocated to each dose level. For each scenario, the true MTD is indicated in bold (probability of DLT = 0.33). All three scenarios are misspecified, so that the results of the simulations do not depend on the logistic model. In scenario one, the MTD is dose level −2. In this case, the probability of recommending this dose level is 0.816, and 58% of the patients are allocated to that level. The average percent of DLT is 42.5%, about 10% higher than the target which is expected when the minimum dose level is the MTD. In the second scenario, the MTD is dose level 0. In this case, the percent of recommendation is 57.3%, and the trial is safe. In scenario three, the MTD is the last dose level. In this case, the percent MTD recommendation is 61.3%, and 93.7% of the trial will recommend either dose level 1 or 2 as the true MTD. Most patients are allocated to the MTD, and the trial is very safe. Based on these scenarios, we conclude that the design performs well with a sample size of 16 patients.

### 3.7. Data Sets Analyzed

All eligible subjects enrolled into the study and who received at least one dose of the study treatment (i.e., the safety population) will be included for safety and preliminary efficacy analyses. All enrolled subjects will undergo peripheral blood draws for plasma metabolomics and, if available, stool collection for microbiome analyses. Those having received the study drug will be included in the exploratory biomarker analyses.

### 3.8. Primary Endpoint Analysis

The primary analysis will be based on all enrolled subjects receiving ≥one dose of study treatment. The primary endpoint analysis of the RP2D will be assessed by the number of DLTs, defined as the rate of treatment-related grade ≥3 AEs occurring within the first 4 weeks (i.e., Cycle 1) of study drugs. The dose level closest to the median of the posterior distribution of the MTD is defined as the RP2D. The dose level such that the probability of DLT at the MTD is *θ* = 0.33 is defined as the MTD.

### 3.9. Secondary Endpoint Analysis

Secondary objectives include safety and preliminary efficacy. All subjects entered into the study who receive any amount of study treatment will be included for safety analysis.

Safety will be assessed by AEs, as summarized by type and severity in the NCI CTCAE, Version 5.0. In particular, AE frequencies by type, severity, body system, and relationship to study treatment will be summarized. SAEs, if any, will be described in detail.

Clinical activity of the combination therapy will be assessed by the objective response rate (ORR), overall survival (OS), and progression-free survival (PFS), which are evaluated every 8 weeks (i.e., every 2 cycles ± 1 week) according to the revised Response Evaluation Criteria in Solid Tumors (RECIST) v1.1 guidelines. Since the goal is to characterize the preliminary therapeutic efficacy of the combination of gemcitabine, nab-paclitaxel, and L-glutamine, no formal hypothesis testing will take place and it will not be based on the power of a test.

### 3.10. Exploratory Endpoint Analysis: Plasma Metabolomics

The exploratory endpoint for the correlative biomarker analyses is the measurement of peak AUCs from a panel of 219 central carbon metabolites that are assessed by mass spectrometry (MS)-based quantitation of collected blood samples from enrolled subjects at the following time points: at baseline (i.e., prior to initiation of the 3–7-day lead-in of L-glutamine); after the 3–7-day lead-in phase with L-glutamine alone; weekly, throughout the first cycle of combination L-glutamine with gemcitabine/nab-paclitaxel; and upon disease progression, intolerance to therapy, or another reason for study discontinuation. Given that the main objective of the correlative studies is to determine the feasibility of longitudinally collecting peripheral blood samples across predefined time points for plasma metabolomics in our study cohort, no formal hypothesis testing will take place and it will not be based on the power of a test.

Plasma metabolites collected at baseline will be correlated with a comprehensive list of clinicopathologic variables, including site of metastases, carbohydrate antigen (CA) 19-9 level, demographics, cachexia, pathologic features, PFS, and OS.

Univariate analyses will be used to assess the association of metabolites with clinicopathologic variables, using parametric or nonparametric tests such as Pearson or Spearman correlation coefficients, the two-sample *t*-test, or the Mann-Whitney test. Preliminary analyses will be carried out to compare changes in metabolites from baseline to each of the time points using paired *t*-tests or the Wilcoxon sign-test, controlling for the proportion of false positives among all significant results, with a false discovery rate (FDR) of 0.1 via the Benjamini and Hochberg procedure, to adjust for multiplicity [66].

### 3.11. Exploratory Endpoint Analysis: Stool Microbiome

The second exploratory endpoint is the relative bacterial abundance assessed by shotgun metagenomics from available stool samples collected from enrolled subjects at baseline (i.e., prior to L-glutamine lead-in phase) and after the L-glutamine lead-in phase, before initiation of gemcitabine/nab-paclitaxel. Shotgun metagenomics will be employed on stool samples with diversity indices (Shannon and Chao1) and the relative abundance at the species level compared across clinicopathologic variables, including cachexia and rates of gastrointestinal side effects.

### 3.12. Collection of Plasma Specimens

All consenting and enrolled patients in this study will need to undergo serial peripheral blood draws during predefined study time points (Table 1 and Figure 1), and under prespecified conditions (i.e., morning, fasted blood collections) for evaluation of changes in plasma metabolites before and after study treatment.

Peripheral blood draws (one, 10 mL K2 EDTA (etheylenediaminetetraacetic acid) Vacutainer^®^ tube per collection, for plasma metabolomics will be collected at the same time of the day (morning time frame of 7–11 a.m.) and in fasting patients (at least 8 h from last meal) at the following study time points: baseline; after a 7-day lead-in phase of L-glutamine administration; weekly, throughout the first cycle of combination L-glutamine with gemcitabine/nab-paclitaxel (including Day 28 of Cycle 1 or Day 1 of Cycle 2); and upon disease progression, intolerance to therapy, or another reason for study discontinuation (Table 1). We expect seven peripheral blood collections, at most, for each subject (including baseline) for metabolomics. All venous blood draws for plasma metabolomics will be collected during standard clinical phlebotomy under the aforementioned prespecified conditions, according to the standard operating protocol (SOP).

### 3.13. Plasma Metabolomics Analyses

For exploratory analyses, the plasma metabolomics assay (Agilent Triple Quad) is coupled to a high-pressure liquid chromatography (HPLC) set up, which has been specifically purposed for MS-based quantitation to perform measurements of the peak area under the curve (AUC) for the metabolites of interest. An automated sample preparation method using the Bravo Metabolomics Sample Prep Platform (Bravo) and Agilent Captiva EMR-Lipid plate will be used to precipitate plasma proteins to quench enzymatic activity, deplete lipids, and extract metabolites, ahead of the liquid chromatography (LC)/MS analysis.

### 3.14. Stool Microbiome Analyses

Stool microbiome analyses will occur immediately upon enrollment of the last subject with post-L-glutamine lead-in stool collected, but will be batched as a single analysis through the University of California Los Angeles (UCLA) Microbiome Core. Shotgun metagenomics will be performed using the NovaSeq™ 6000 Sequencing System by the UCLA Microbiome Core (Los Angeles, CA, USA).

## 4. Regulatory Oversight

This clinical trial has been approved by the CSMC IRB (protocol code STUDY00001113 and date of approval 23 March 2021). Oversight by the PI is required throughout the entire registration process. All data will be entered into an HIPAA-compliant database. The study staff, in accordance with procedural documentation, will be responsible for data processing. The PI will ensure adherence to the protocol, Good Clinical Practices (GCP), and institutional policy throughout the study duration via routine Disease Research Group (DRG) meetings or equivalent. In addition, the SOCCI Clinical Trials Office Quality Management Core (QMC) will conduct internal monitoring visits and audits for data quality and protocol adherence, along with the SOCCI DSMC.

Oversight of the progress and safety of the study will be provided by the PI. It is the responsibility of the principal investigator to adhere to the Data Safety Monitoring Plan throughout the life of the study.

In addition, the SOCCI DSMC will provide another layer of data and safety oversight. DSMC membership and responsibilities are governed by the committee charter. Every six months, the DSMC recommendations and findings will be reported in writing to the PI as a summary letter, which will then be forwarded by the PI or designee to the CS-IRB. The DSMC may increase or decrease the frequency of study review, at their discretion.

Source documents include all observations recorded or clinical activity notations and all records and reports necessary for the reconstruction and evaluation of the study. The study investigator must retain all source documentation related to the conduct of the clinical trial, as required by government agency regulations and directives. Study documents will be kept on file, as per local guidelines.

## 5. Discussion

Gemcitabine and nab-paclitaxel is currently a standard first-line therapy in treating advanced PDAC. Optimizing the efficacy of first-line therapy is of high unmet need, given the dismal overall prognosis and precipitous decline in receipt of second- and third-line chemotherapy regimens in this patient population [2,3]. Thus, in the GlutaPanc trial, an open-label, single-arm, prospective phase I trial, we investigate the combination L-glutamine, gemcitabine, and nab-paclitaxel serving as an augmented therapeutic strategy in treatment-naïve, unresectable or metastatic pancreatic cancer.

Although initial efforts to target the *Kras*-driven metabolic dependency of PDAC through glutamine antagonism in preclinical models have largely failed [7], preclinical data has shown that direct glutamine supplementation, rather than glutamine deprivation, can elicit antitumor effects in PDAC [8,9,10,11,13]. Based on the *KRAS*-driven glutamine dependency of PDAC, we will measure the effects of L-glutamine supplementation on various metabolic pathways, including the mitochondrial oxidative phosphorylation, nonoxidative pentose phosphate, hexosamine biosynthesis, amino acid metabolism, and lipid metabolism, to gain mechanistic insights and explore potential metabolic biomarkers for this novel therapy [4,5,6].

Over the past three decades, enteral glutamine supplementation has been investigated in adult and pediatric patients with cancer treated with radiotherapy, chemotherapy, and stem cell transplantation as a means of mitigating mucositis, neuropathy, and radiation enteritis [16,21,22,23,24,25,26,27,28,29,30,31,32,33,34,35,36,37,38,39,40,41,42,43,44,45,46,47,48,49,50,51,52,53,54,55,56,57,58,59,62,63,64]. Other studies on glutamine intake have supported improvements in protein balance in tumor-bearing animals, enhancements in the function of natural killer cells, and improvements in overall immunity and gut integrity with chronic glutamine supplementation [67].

In an earlier study, six healthy male subjects were administered 0, 0.1, or 0.3 g/kg of oral glutamine weekly with three doses, and blood samples were collected at multiple time points after glutamine administration [64]. Oral glutamine resulted in significant increases in amino acid concentrations known to be the end-products of glutamine metabolism, including arginine, citrulline, alanine, and glutamate. On the other hand, as the glutamine dose increased, levels of glycine, methionine, phenylalanine, leucine, and branched-chain amino acids decreased.

By collecting fasted blood samples before and after a 1-week L-glutamine alone lead-in, followed by serial collections of blood samples upon the introduction of gemcitabine and nab-paclitaxel, we are well-positioned to evaluate the resultant metabolomic profiles from L-glutamine and the combination strategy in this study. This serves as a key strength of this study, as conducting plasma metabolomics in our cohort can provide us with potential mechanistic insights regarding the anticancer properties of L-glutamine, which may rely on the previously described metabolic pathways altered by mutant *KRAS*. From the plasma metabolomics, we aim to generate further hypotheses for future research on potential associations between metabolic profiles associated with the therapeutic response (or lack thereof). Moreover, these plasma metabolites may potentially serve as useful biomarkers to objectively measure patient response to treatment, particularly for chemotherapy and glutamine.

Additionally, another strength of the study is the measurement of glutamine supplementation’s impact on the gut microbiome of human PDAC subjects before and after the L-glutamine lead-in phase. Because L-glutamine has been shown to promote gut health [14], L-glutamine supplementation may prevent or reduce the rate of gastrointestinal toxicities from chemotherapy. By analyzing the abundance of gut microbiome from available stool samples, we may associate an improvement in GI toxicities with changes to the gut microbiome composition resulting from the study therapy. Modulation of the microbiome has also been implicated in influencing therapeutic responses to cancer therapy and cachexia, the latter being highly prevalent and a cause of significant morbidity in patients with PDAC [68,69]. Thus, our microbiome analyses will provide much needed insight into the separate mechanisms by which L-glutamine supplementation may confer clinical benefit.

The study has a few limitations and challenges. One potential limitation is the relatively small sample size of the study. However, our sample size was designed to rapidly accrue and identify the MTD of L-glutamine and combination chemotherapy, with preliminary efficacy as a secondary objective. As described in the Methods and Materials section, upon enrollment of a total of 16 patients into the DLT assessment cohort, RP2D will be assessed by EWOC, an adaptive Bayesian design [17], using the dosing schema proposed in Table 5.

A potential challenge to study accrual and maintaining subjects on study is the relatively poor solubility of L-glutamine in water, which subsequently requires relatively large quantities of oral liquid intake each day. This fact may discourage the adherence to the daily glutamine intake as described in the study protocol. The difficulty with adherence may be further exacerbated if the clinical status of the patients deteriorates over time. Nevertheless, the 7-day lead-in phase with L-glutamine should aid in alleviating some of the compliance concerns, as oral compliance for study continuation is considered >85%. This will allow us to screen and identify subjects who may experience difficulty in tolerating oral glutamine during the study. Ultimately, given the phase I design of this study, further prospective validation of the efficacy of this combination will be needed in larger phase II and III settings to advance this combination as a potential new, standard option. The attractiveness of L-glutamine supplementation remains in its relatively tolerable safety profile, given that it is widely available as a dietary supplement. Furthermore, we have experience with L-glutamine supplementation over the past three decades, from its use in the prevention of radiotherapy and chemotherapy side effects in patients with various malignancies. We therefore expect the addition of L-glutamine to nab-paclitaxel and gemcitabine to be safe and well tolerated. If an early efficacy signal could be shown from this phase I trial, we will be primed to advance this combination as a novel strategy to target the glutamine dependency of PDAC, when efforts over the past few decades have relied on glutamine deprivation as the fundamental approach to target PDAC tumor metabolism.

## 6. Conclusions

The phase I GlutaPanc will investigate a novel therapeutic strategy incorporating the only FDA-approved, therapeutic-grade L-glutamine with standard chemotherapy gemcitabine and nab-paclitaxel in the first-line treatment of metastatic PDAC. This approach is novel, given that historically, the antagonism of glutamine signaling (i.e., glutamine deprivation) has been explored to target the glutamine dependency of PDAC. Based on preclinical evidence that L-glutamine supplementation, rather than deprivation, elicits anticancer effects, we propose to evaluate the feasibility of adding glutamine to standard chemotherapy. We hypothesize that the addition of L-glutamine to standard chemotherapy is safe at maximum standard doses of chemotherapy. We would also be among the first to perform serial plasma metabolomics and evaluate the stool microbiome composition which are resultant from L-glutamine therapy. These pre-planned exploratory analyses would allow the evaluation of changes in multiple metabolic pathways that may be altered by L-glutamine, in order to identify potential mechanistic insights and associations with study treatment, while facilitating the analysis of L-glutamine’s impact on promoting gut health as an additional potential mechanism of benefit. If positive, we would seek to advance the combination therapy through a randomized trial of patients with metastatic pancreatic cancer in the first-line setting, a high-risk subgroup desperately in need of additional therapies that can improve upon the dismal outcomes seen in this population.

## Figures and Tables

**Table 1:** Standard Protocol Items for Intervention Trials (SPIRIT): schedule of enrollment, interventions, and assessments.

Procedure	Screening/Baseline	Treatment Phase until Progression, Intolerance, or Withdrawal		End of Treatment	Follow-Up
	All to Occur within 4 Weeks Prior to the Lead-In Week of L-Glutamine or within 5 Weeks of Cycle 1 Day 1	Day 1, 8, 15, 28 (±3 Days) of Cycle 1 L-Glutamine, Gemcitabine, and Nab-Paclitaxel	Day 8, 15 (±3 Days) of Cycle 2 and Day 1, 8, 15 (±3 Days) of Cycle 3 Onward	Within 30 Days after Study Termination (±7 Days)	Survival Follow-Up Every 3 Months for 24 Months (±7 Days)
Informed Consent ^a^	X				
Eligibility review	X				
Medical history review ^b^	X				X
Demographics	X				
Performance Status (ECOG/KPS) ^c^	X	X	X	X	
Concomitant medications	X	X	X	X	
Complete Physical Exam	X	X	X	X	
Height	X				
Weight	X	X	X	X	
Vital Signs ^d^	X	X	X	X	
Adverse Event Assessment ^e^	X	X	X	X	
Blood collection (CBC, CMP)	X	X	X	X	
Pregnancy test ^f^	X				
Plasma metabolomics ^g^	X	X		X	
Tumor assessment (imaging) ^h^	X		X ^h^ (every 2 cycles)		
Tumor marker (CA 19-9) ^i^	X		X	X	
Drug diary review ^j^		X	X	X	
L-glutamine ^k^		X	X		
Gemcitabine ^l^		X	X		
Nab-paclitaxel ^l^		X	X		
Survival Follow-up ^m^					X
Stool microbiome ^n^	X				

^a^ Informed consent must be obtained within 6 weeks of Cycle 1 Day 1; ^b^ Relevant medical history, including history of current disease, surgical history, and information regarding underlying diseases will be recorded at Screening; ^c^ According to Eastern Cooperative Oncology Group (ECOG) criteria or Karnofsky Performance Status (KPS) scale; ^d^ Vital signs to include temperature, pulse, respirations, blood pressure; ^e^ Safety evaluation as per Common Terminology Criteria for Adverse Events (CTCAE) version 5.0; dose-limiting toxicity (DLT) assessment window is first cycle (4 weeks);^f^ Urine or serum pregnancy test within 14 days prior to receiving the first dose of study medication for eligibility verification. If the urine test is positive or cannot be confirmed as negative, a serum pregnancy test will be required. Note: Negative urine or serum pregnancy test is also conducted within 72 h prior to Cycle 1 Day 1 (C1D1) for study procedures, and should not preclude eligibility verification, but if screening pregnancy test is done within 72 h of C1D1, it is not required to be repeated; ^g^ Collected at the same time of the day (morning timeframe of 7–11 a.m.) and in fasting patients (at least 8 h from last meal) at baseline, after a 1-week administration of L-glutamine, weekly throughout the first cycle of combination gemcitabine/nab-paclitaxel and L-glutamine (including Day 28 of Cycle 1 or Day 1 of Cycle 2), and upon disease progression, intolerance to therapy, or other reason for study discontinuation. We expect at most 7 peripheral blood collections for each subject (including baseline) for metabolomics; ^h^ Evaluation of tumor response (every 8 weeks, as per standard of care, ±7 days) according to Response Evaluation Criteria in Solid Tumors (RECIST) v1.1; ^i^ Every 28 days from Cycle 1 Day 1; ^j^ All subjects will be provided with instructions and drug diary (oral drug compliance < 85% is considered a deviation that is reportable to the Institutional Review Board [IRB]); ^k^ L-glutamine will be administered alone for 3–7 days prior to addition of gemcitabine/nab-paclitaxel. L-glutamine will be given as 0.1, 0.2, or 0.3 g/kg oral twice daily (rounded to nearest 5 g) with an upper limit of 15 g twice daily (30 g/day) for any dose level; ^l^ Gemcitabine and nab-paclitaxel doses are assigned. Treatment can continue even after completion of study, as per investigator’s discretion; ^m^ Survival follow-up includes medical chart review for survival. If no records of patient survival in medical chart, the principal investigator (PI) or study staff may call to assess survival; ^n^ Collected at baseline or prior to initiation of L-glutamine lead-in and upon completion of glutamine lead-in, but prior to initiation of chemotherapy, if available.

**Table 2:** Inclusion criteria for the GlutaPanc trial.

Advanced or unresectable, histologically confirmed pancreatic cancer (new diagnosis or recurrent, where recurrent tumor does not need to be histologically-confirmed) referred to CSMC SOCCI for first-line chemotherapy. Prior neoadjuvant or adjuvant chemotherapy and/or chemoradiation is allowed, but must have been completed ≥6 months prior to recurrence.
Age ≥ 18 years
ECOG performance status ≤2 or Karnofsky performance status ≥60%
Demonstrate adequate organ and marrow function as defined below (within 14 days of study treatment initiation): Hematologic: ANC (≥1500/μL), platelets (≥100,000/μL), hemoglobin (≥9 g/dL or ≥5.6 mmol/L) Renal: Serum creatinine or PoCT creatinine (≤1.5 X ULN) OR measured or calculated creatinine clearance ≥45 mL/min/1.73 m^2^ for subject with creatinine levels > 1.5 X institutional ULN) Hepatic: Serum total bilirubin (≤1.5 X ULN OR direct bilirubin ≤ ULN for subjects with total bilirubin levels > 1.5 ULN), AST/SGOT and ALT/SGPT (≤5 X ULN)
Have measurable disease, based on RECIST 1.1
Female subject of childbearing potential should have a negative urine or serum pregnancy within 14 days prior to receiving the first dose of study medication for eligibility verification. If the urine test is positive or cannot be confirmed as negative, a serum pregnancy test will be required. Note: negative urine or serum pregnancy test is also conducted within 72 h prior to C1D1 for study procedures, and should not preclude eligibility verification, but if screening pregnancy test is done within 72 h of C1D1, it is not required to be repeated.
Female subjects of childbearing potential should be willing to use adequate methods of birth control (hormonal or barrier method of birth control) or be surgically sterile, or abstain from heterosexual activity for the course of the study through 120 days after the last dose of study medication. Subjects of childbearing potential are those who have not been surgically sterilized or have not been free from menses for >1 year.
Male subjects should agree to use an adequate method of contraception, starting with the first dose of study therapy through 120 days after the last dose of study therapy.
Willingness to undergo serial peripheral blood draws and provide stool samples during predefined study timepoints and under prespecified conditions (i.e., fasted, morning blood collections).
Written informed consent obtained from subject and ability for subject to comply with the requirements of the study.

Abbreviations: CSMC, Cedars-Sinai Medical Center; SOCCI, Samuel Oschin Comprehensive Cancer Center; ECOG, Eastern Cooperative Oncology Group; ANC, absolute neutrophil count; PoCT, point-of-care testing; ULN, upper limit of normal; RECIST, response evaluation criteria in solid tumors; C1D1, cycle 1 day 1.

**Table 3:** Exclusion criteria for the GlutaPanc trial.

Is currently participating in, or has participated in, a study of an investigational agent or using an investigational device within 4 weeks of the first dose of treatment.
Has previously received chemotherapy for metastatic disease (treatment with neoadjuvant or adjuvant therapy is allowed, so long as treatment was completed ≥6 months prior to recurrence).
Has pre-existing grade ≥3 neuropathy.
Has a known additional malignancy that is progressing or requires active treatment. Exceptions include basal cell carcinoma of the skin, squamous cell carcinoma of the skin, or in situ cervical cancer that has undergone potentially curative therapy.
Has a known hypersensitivity to any components of the study drugs.
Has known active central nervous system metastases and/or carcinomatous meningitis. Subjects with previously treated brain metastases may participate provided they are stable (without evidence of progression by imaging for at least four weeks prior to the first dose of trial treatment, and any neurologic symptoms have returned to baseline), have no evidence of new or enlarging brain metastases, and are not using steroids for at least seven days prior to trial treatment.
Has a history or current evidence of any condition, therapy, or laboratory abnormality that might confound the results of the trial, interfere with the subject’s participation for the full duration of the trial, or make it not in the best interest of the subject to participate, in the opinion of the treating investigator.
Has known psychiatric or substance abuse disorders that would interfere with cooperation with the requirements of the trial.
Is pregnant or breastfeeding or expecting to conceive or father children within the projected duration of the trial, starting with the pre-screening or screening visit through 120 days after the last dose of trial treatment.
Has any gastrointestinal disorder (e.g., bowel obstruction) or neurologic condition (e.g., oropharyngeal dysphagia) that may result in impairment of oral intake and/or absorption of study drug in the opinion of the treating investigator.
Is currently receiving any parenteral nutrition or enteral (tube) feeding or is planning to use any other nutritional supplement (defined as any other nutritional supplement containing *any* amount of glutamine) during the study period.
Patients on strong CYP2C8 or CYP3A4 inhibitors or inducers within 1 week prior to starting nab-paclitaxel.

**Table 4:** Screening Procedures of the GlutaPanc trial.

Informed consent	Must be obtained within 6 weeks of Day 1.
Medical history and record review	Relevant medical history, including history of current disease, surgical history, and information regarding underlying diseases will be recorded at screening.
Demographics	Age, gender, race, ethnicity.
Subject eligibility criteria review	
Concomitant medications	
Physical exam	Vital signs (temperature, pulse, respirations, blood pressure), height, and weight.
Performance status	Evaluated prior to study entry according to ECOG criteria or KPS scale.
Adverse event assessment	Baseline adverse events will be assessed from C1D1 until the End of Treatment Visit (within 30 days of treatment discontinuation ±7 days). Duration (start and stop dates and times), severity/grade, outcome, treatment and relation to study drug will be recorded on the CRF.
Laboratory evaluations	CBC with differential, CMP.
Pregnancy testing for WOCBP	Urine or serum pregnancy required within 14 days prior to receiving first dose of study medication for eligibility. If urine test is positive or cannot be confirmed as negative, then a serum pregnancy test is required. For safety, a negative pregnancy test is required within 72 h of first dose but does not preclude eligibility verification. Note: if screening pregnancy test is done within 72 h of C1D1, it is not required to be repeated.
Blood draw for tumor assessment	Carbohydrate antigen (CA) 19-9 will be assessed.
Blood draw for plasma metabolomics	Obtained along with standard of care laboratory evaluations (under fasted, morning blood collection) for baseline plasma metabolomics.
Stool sample for microbiome analyses	Obtained prior to initiation of L-glutamine lead-in and upon completion of glutamine lead-in, but prior to initiation of chemotherapy, if available.
CT imaging/scan	Baseline evaluation of measurable disease per RECIST v1.1.

Abbreviations: ECOG, Eastern Cooperative Oncology Group; KPS, Karnofsky Performance Status; C1D1, cycle 1 day 1; CRF, case report form; CBC, complete blood count; CMP, complete metabolic panel; RECIST, response evaluation criteria in solid tumors.

**Table 5:** Dosing Levels ^a^.

Dose Level	Gemcitabine	Nab-Paclitaxel	L-Glutamine ^b^
−2	600 mg/m^2^ IV D1, 8, 15	75 mg/m^2^ IV D1, 8, 15	0.1 g/kg oral BID ^c^
−1	800 mg/m^2^ IV D1, 8, 15	100 mg/m^2^ IV D1, 8, 15	0.1 g/kg oral BID
0	1000 mg/m^2^ IV D1, 8, 15	125 mg/m^2^ IV D1, 8, 15	0.1 g/kg oral BID
1	1000 mg/m^2^ IV D1, 8, 15	125 mg/m^2^ IV D1, 8, 15	0.2 g/kg oral BID
2	1000 mg/m^2^ IV D1, 8, 15	125 mg/m^2^ IV D1, 8, 15	0.3 g/kg oral BID

^a^ Every 28-day cycle, ^b^ Rounded to nearest 5 g, upper limit of 15 g twice daily (30 g/day) for any dose level; started 1 week (±1 day) prior to chemotherapy, ^c^ BID: twice a day.

**Table 6:** Dose Level Reductions for Treatment-related Adverse Events from Gemcitabine and Nab-paclitaxel.

Dose Level ^a^	Nab-Paclitaxel (mg/m^2^)	Gemcitabine (mg/m^2^)
Full dose	125	1000
1st dose reduction	100	800
2nd dose reduction	75	600
If additional dose reduction required	Discontinue	Discontinue

^a^ Dose reductions should occur with reference to starting dose level, e.g., if starting at 100 mg/m^2^ and 800 mg/m^2^ of nab-paclitaxel and gemcitabine, respectively, next dose reduction would be 75 mg/m^2^ and 600 mg/m^2^ level, respectively.

**Table 7:** Dose Modifications for Hematologic Toxicities from Gemcitabine and Nab-paclitaxel.

Cycle Day	ANC (cells/mm^3^)		Platelet Count (cells/mm^3^)	Gemcitabine/Nab-Paclitaxel
Day 1	<1500	OR	<100,000	Delay dose until recovery
Day 8	500 to <1000	OR	50,000 to <75,000	Reduce 1 dose level
	<500	OR	<50,000	Withhold dose
Day 15: IF Day 8 doses were reduced or given without modification:				
	500 to <1000	OR	50,000 to <75,000	Reduce 1 dose level from Day 8
	<500	OR	<50,000	Withhold dose
Day 15: IF Day 8 doses were withheld:				
	≥1000	OR	≥75,000	Reduce 1 dose level from Day 1
	500 to <1000	OR	50,000 to <75,000	Reduce 2 dose levels from Day 1
	<500	OR	<50,000	Withhold dose

**Table 8:** Dose Modifications for Other Toxicities from Gemcitabine and Nab-paclitaxel.

Treatment-Related AE	Nab-Paclitaxel	Gemcitabine
Febrile Neutropenia: Grade 3 or 4	Withhold until fever resolves and ANC ≥ 1500; resume at next-lower dose level	
Peripheral Neuropathy: Grade 3 or 4	Withhold until improves to ≤grade 1; resume at next-lower dose level	No dose reduction
Cutaneous Toxicity: Grade 2 or 3	Reduce to next-lower dose level; discontinue treatment if toxicity persists	
Gastrointestinal Toxicity: Grade 3 mucositis or diarrhea	Withhold until improves to ≤grade 1; resume at next-lower dose level	
Other Non-hematologic Toxicity: Grade 3 or 4	Withhold until improves to ≤grade 1; resume at next-lower dose level (no dose modifications recommended for alopecia, nausea, or vomiting)	Withhold or reduce dose by 50% until resolved (no dose modifications recommended for alopecia, nausea, or vomiting)

**Table 9:** Reasons for removal of subjects from GlutaPanc trial.

Patient voluntarily withdraws (follow-up permitted).
Patient withdraws consent (termination of treatment and follow-up).
Patient is unable to comply with protocol requirements.
Patient demonstrates disease progression (unless continued treatment with study drug is deemed appropriate, at the discretion of the investigator).
Patient experiences toxicity that makes continuation in the protocol unsafe.
Patient is unable to finish a minimum of 3 days of L-glutamine during 3–7-day lead-in.
Treating physician determines continuation on the study would not be in the patient’s best interest.
Patient becomes pregnant (pregnancy to be reported along same timelines as a serious adverse event).
Development of second malignancy (except for basal cell carcinoma or squamous cell carcinoma of the skin) that requires treatment, which would interfere with this study.
Lost to follow-up. If a research subject cannot be located to document survival after a period of 2 years, the subject may be considered “lost to follow-up.” All reasonable efforts must be made to locate subjects, to determine and report their ongoing status. Lost to follow-up is defined by the inability to reach the subject after a minimum of three documented phone calls, faxes, or emails, as well as lack of response by subject to one registered-mail letter. All attempts should be documented in the subject’s medical records. If it is determined that the subject has died, the site will use permissible local methods to obtain the date and cause of death. If, after all attempts, the subject remains lost to follow-up, then the last-known alive date, as determined by the investigator, should be reported and documented in the subject’s medical records.

**Table 10:** Design operating characteristics for safety and efficiency (true MTD in bold).

	Dose Level				
Scenario 1	**−2**	−1	0	1	2
True Prob DLT	**0.33**	0.45	0.65	0.75	0.90
%MTD recommendation	**81.6**	17.7	7.0	0.0	0.0
% patients allocated	57.9	26.1	12.1	3.3	0.6
Ave % DLTs	42.5				
% Trials: rate DLT > *θ* + 0.1	57.5				
	Dose Level				
Scenario 2	−2	−1	**0**	1	2
True Prob DLT	0.01	0.05	**0.33**	0.50	0.70
%MTD recommendation	0.0	28.0	**57.3**	13.9	0.8
% patients allocated	2.5	25.5	46.1	18.4	7.2
Ave % DLTs	30.1				
% Trials: rate DLT > *θ* + 0.1	5.1				
	Dose Level				
Scenario 3	−2	−1	0	1	**2**
True Prob DLT	0.001	0.03	0.08	0.15	**0.33**
%MTD recommendation	0.00	0.20	0.61	32.4	**61.3**
% patients allocated	0.50	2.73	14.95	27.37	54.42
Ave % DLTs	23.08				
% Trials: rate DLT > *θ* + 0.1	0.50				

Abbreviations: MTD, maximum tolerated dose; DLT, dose-limiting toxicity; Ave, average.

## Data Availability

The trial sponsor (Emmaus) and the conducting institution will have access to the final trial dataset. Requests for access will be granted solely at the discretion of the sponsor and PI. Access to full protocol and datasets will be reviewed and granted at the discretion of the sponsor and PI. The authors plan to present results at a national cancer conference and publish final results in a peer-reviewed journal.

## Abbreviations

PDAC: pancreatic ductal adenocarcinoma; RP2D: recommended phase II dose; 5-FU: 5-fluorouracil; FOLFIRINOX: leucovorin, 5-fluorouracil, irinotecan, and oxaliplatin; PS: performance status; TCA: tricarboxylic acid; ATP: adenosine triphosphate; NADPH: reduced nicotinamide adenine dinucleotide phosphate; NADP+: nicotinamide adenine dinucleotide phosphate; IKKβ: IκB kinase β; ROS: reactive oxygen species; ECOG: Eastern Cooperative Oncology Group; KPS: Karnofsky Performance Status; CTCAE: Common Terminology Criteria for Adverse Events; DLT: dose-limiting toxicity; C1D1: cycle 1 day 1; RECIST: Response Evaluation Criteria in Solid Tumors; IRB: Institutional Review Board; PI: principal investigator; CSMC: Cedars-Sinai Medical Center; SOCCI: Samuel Oschin Comprehensive Cancer Center; ANC: absolute neutrophil count; PoCT: point-of-care testing; ULN: upper limit of normal; CRF: case report form; CBC: complete blood count; CMP: complete metabolic panel; MTD: maximum tolerated dose; EWOC: escalation with overdose control; IV: intravenous; AE: adverse event; BID: twice a day; FDA: Food and Drug Administration; PD: progressive disease; NCI: National Cancer Institute; EOT: end of treatment; EDTA: etheylenediaminetetraacetic acid; SOP: standard operating protocol; CIOMS: Council for International Organizations of Medical Sciences; SAE: serious adverse event; DSMC: Data and Safety Monitoring Committee; CCTO: Cancer Clinical Trials Office; HPLC: high-pressure liquid chromatography; MS: mass spectrometry; AUC: area under the curve; LC: liquid chromatography; UCLA: University of California Los Angeles; ANOVA: analysis of variance; ANCOVA: analysis of covariance; ORR: objective response rate; PFS: progression-free survival; OS: overall survival; CA: carbohydrate antigen; FDR: false discovery rate; QMC: Quality Management Core; HIPAA: Health Insurance Portability and Accountability Act; GCP: Good Clinical Practices; DRG: Disease Research Group.
